# Unravelling the TL1A-DR3 axis in Hidradenitis suppurativa

**DOI:** 10.3389/fimmu.2026.1837120

**Published:** 2026-04-22

**Authors:** Juling Sia, Bernett Lee, Hazel H. Oon

**Affiliations:** 1 Lee Kong Chian School of Medicine, Nanyang Technological University, Singapore, Singapore; 2 Singapore Immunology Network (SigN), Agency for Science, Technology and Research (ASTAR), Singapore, Singapore; 3 Infectious Disease Labs (ID Labs), Agency for Science, Technology and Research (ASTAR), Singapore, Singapore; 4 Khoo Teck Puat Hospital, Singapore, Singapore; 5 National Skin Centre and Skin Research Institute of Singapore, Singapore, Singapore; 6 Yong Loo Lin School of Medicine, National University of Singapore, Singapore, Singapore

**Keywords:** biologics, DR3, Hidradenitis suppurativa, inflammation, TL1A, afimkibart, duvatikug, fibrosis

## Abstract

Hidradenitis suppurativa (HS) is a chronic inflammatory disorder that presents with both cutaneous manifestations and extracutaneous comorbidities. Current treatment options include antibiotics and biologics; however, treatment failure is common and often surgical intervention is required for acceptable control. Anti-TL1A therapies have recently gained attention in HS treatment due to their ability to inhibit the TL1A/DR3 axis, a key signaling pathway driving immune cell activity and fibrosis. Participant recruitment for the phase 2b trial (NCT06956235) assessing the efficacy of anti-TL1A therapy tulisokibart in moderate-to severe HS was reported on 1 December, 2025 to be completed. This review aims to explore the potential links between the TL1A and HS by synthesizing immune mechanisms of HS pathogenesis with existing data of the TL1A/DR3 axis in immune pathways. A discussion highlighting the potential for anti-TL1A therapies as a class that simultaneously addresses fibrosis and comorbidities of HS is also presented, and future directions to address knowledge gaps are also proposed.

## 1 Introduction

Hidradenitis suppurativa (HS) is a chronic inflammatory skin disorder that is characterized by the presence of recurrent painful abscesses, nodules and draining sinuses in intertriginous areas such as the axilla and groin (1, 2). Clinical manifestations typically commence between the second to fourth decades of life, with affected individuals frequently exhibiting a concomitant spectrum of cardiometabolic and autoimmune comorbidities (3, 4). Suboptimal control of HS can have profound implications on quality of life, including chronic pain, poor mental health, cosmetic concerns and financial strain from frequent medical visits (3, 4).

Current management guidelines for HS primarily address its immune-mediated and bacterial pathophysiology, including the role of biofilms in follicular occlusion and chronic suppuration. First-line therapy typically consists of antibiotics alongside symptomatic measures (1). In more severe disease, escalation to biologic therapy such as adalimumab, secukinumab and bimekizumab may be pursued (5), However, these therapies do not consistently achieve long-term remission and are associated with variable response rates (1, 6, 7). Patients often require a combination of pharmacological and surgical therapy for adequate disease control (5, 6). Nevertheless, surgical interventions carry procedural risks, including postoperative infection, to which HS patients are particularly predisposed, as well as risk of recurrence (1, 6–8).

Tumor necrosis factor-like cytokine 1A (TL1A) is a tumor necrosis factor (TNF) superfamily protein that can be expressed in both soluble and membrane-bound forms (9, 10). It is thought to play a central role in the progression of various inflammatory diseases, with previous clinical trials demonstrating anti-TL1A therapies such as tulisokibart, afimkibart, and duvakitug to be effective in the treatment of inflammatory bowel disease (IBD) (10–12). These emerging anti-TL1A therapies are monoclonal antibody therapies that selectively inhibit TL1A activity, and a phase 2b randomized study to evaluate the efficacy and safety of tulisokibart in moderate-to-severe HS is currently ongoing (NCT06956235).

**Figure 1:**
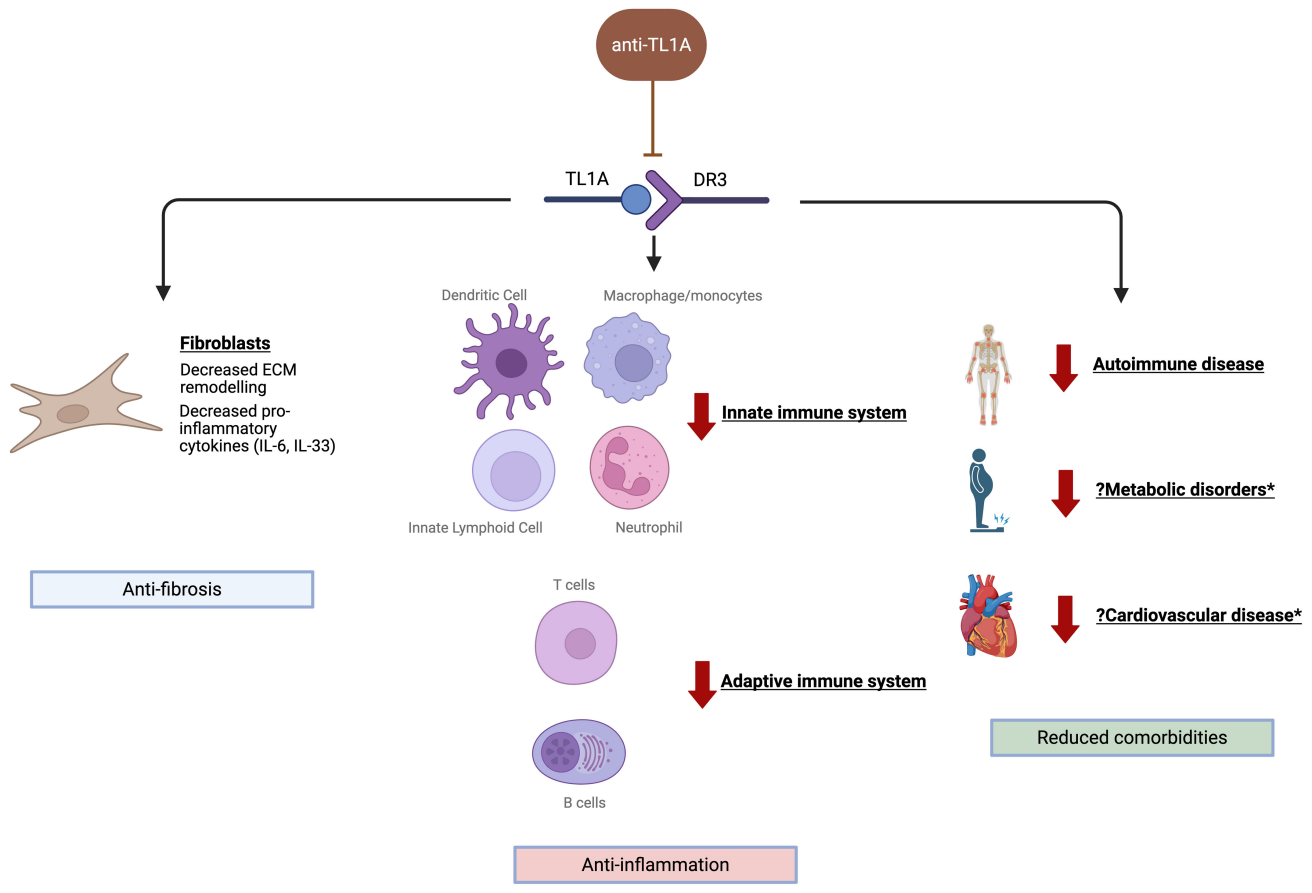
Summary of a proposed triple-pronged approach targeting anti-fibrotic, anti-inflammatory and reduced comorbidity mechanisms by which anti-TL1A therapies may attenuate HS pathogenesis. Created in BioRender. Tey, H. L. (2026) https://BioRender.com/n7kl1g0. *Proposed mechanism is based on pre-clinical evidence. ECM, extracellular matrix; TL1A, tumor necrosis factor-like cytokine 1A; DR3, death receptor 3.

Although TL1A has been directly implicated in several inflammatory diseases such as rheumatoid arthritis and IBD, data specific to HS and its mechanistic underpinnings are only beginning to emerge. Accordingly, this review evaluates the emerging role of anti-TL1A therapy in HS management and advances a triple-pronged framework that extends beyond conventional thinking of anti-TL1A treatment as solely immunosuppressive interventions (**Figure 1**). These insights will be crucial for contextualizing and corroborating findings with ongoing trials, and for informing the design of future clinical studies in the field. In addition, this review seeks to delineate research gaps and provide focused recommendations to guide subsequent research.

## 2 The conventional approach addressing inflammation

TL1A is expressed by both immune and non-immune cells, including dendritic cells (DCs), macrophages, fibroblasts and endothelial cells (13–15). This membrane-bound version of TL1A may subsequently be cleaved by matrix metalloproteinases to yield soluble TL1A; both membrane-bound and soluble TL1A promote inflammatory activity by binding to death receptor 3 (DR3), a transmembrane protein expressed by both adaptive and innate immune cells, such as T cells, B cells and innate lymphoid cells (ILCs), and on TL1A-expressing cells such as macrophages (9, 16–18).

Although the pathogenesis of HS has yet to be fully elucidated (19), converging evidence supports a multifactorial model involving dysregulated innate and adaptive immune response, with follicular occlusion and microbial dysbiosis (2). Given the broad-reaching effects of the TL1A-DR3 axis on various immune cells, we propose that coordinated inhibition of pathogenic adaptive and innate immune pathways is the first mechanism by which anti-TL1A therapies attenuate HS.

### 2.1 Adaptive immune system

Earlier work has suggested that HS development is driven predominantly by an expansion of specific T cell subsets, particularly T helper cells (Th) 1 and 17 (20, 21). Multiple studies have demonstrated that skin from HS patients contained higher levels of Th17 inflammatory cytokines compared to healthy controls (22, 23). A study by Kelly et al. (2015) on 44 patients showed that lesional and perilesional HS biopsies are enriched with IL-17-producing CD4+ T cells in the diseased skin, supporting a central role for Th17 in the disease pathology (22). Similarly, HS pathogenesis has been linked with upregulated Th1 activity, and Thomi et al. (2018) found that HS lesions had increased expression of both Th1 and Th17 cytokines (24–26).

Aberrant regulatory T cell (Treg) function, typically responsible for limiting excessive immune activation, has also been implicated in HS (27), although existing findings remain somewhat inconsistent. Hessam et al. (2020) reported that HS patients had decreased proportions of circulating Tregs in peripheral blood but no significant difference in Treg-derived cytokines such as IL-10 (28). Moran et al. (2017) demonstrated that HS skin biopsies had increased absolute numbers of Tregs, but the proportion of Tregs was reduced compared to Th17, indicating a relative insufficiency of Tregs (20).

Emerging evidence in recent years also suggests that B cells contribute to HS. Analysis of skin samples from 49 HS patients by Abu Rached et al. (2025) showed that a substantial portion (33.3%) of the samples were dominated by B instead of T cells (27). Transcriptome analysis by Gudjonsson et al. (2020) also supports this; apart from elevated levels of IL-17A protein suggestive of a Th17-skewed response, they found that the predominant leukocyte population in lesional skin from 22 patients with moderate-to-severe HS consisted of plasma cells followed by B cells (29). An abstract presented at the 53^rd^ annual European Society for Dermatological Research meeting also reported that oral Bruton’s tyrosine kinase (BTK) inhibitor remibrutinib reported superior HiSCR response rates in HS compared to the placebo, thus underscoring the importance of the BTK/B cell signaling axis (30).

Inhibiting the TL1A/DR3 axis with anti-TL1A therapy could restore this dysregulation of the adaptive immune system in HS. TL1A is known to induce expansion of Th1 and Th17 (9, 13). Meng et al. (2023) found that TL1A-deficient mice had reduced Th1 and Th17 differentiation and did not develop colonic inflammation (31). Taraban et al. (2011) also found that TL1A-induced Treg proliferation in mice but paradoxically diminished individual Treg ability (32). TL1A blockade may therefore reduce HS progression by reducing pathogenic Th1/Th17 and enhancing Treg activity.

There is also evidence to suggest that TL1A can influence B cell and plasma cell activity. Although early *in vitro* work showed that TL1A reduced B cell proliferation induced by anti-IgM/IL-2, more recent studies have demonstrated that TL1A can support plasma cell survival and enhance antibody production (33, 34). In mice with allergic airway disease, anti-TL1A therapy reduced the number of lung plasma cells, an effect attributed to either direct interference of the TL1A/DR3 axis or indirectly via reduction of TL1A-induced IL-9 production (35).

### 2.2 Innate immune system

Innate immune cells have likewise been linked to HS pathogenesis. In both perilesional and lesional skin from HS patients, DCs represent one of the predominant infiltrating myeloid populations (36, 37). DCs promote inflammation in HS by producing chemotactic pro-inflammatory cytokines such as TNF-α (38). They also produce other cytokines, such as IL-23, that promote the differentiation of naïve T cells into Th17, another key mediator of HS development (37). DC-derived cytokines such as TNF-α, IL-23 and IL-1β, are elevated in both skin and serum samples of HS patients (39–41), further supporting a pathogenic role for DC-driven cytokines in HS pathogenesis.

The role of distinct ILC subsets is an additional pathway in HS pathogenesis. According to Petrasca et al. (2023), lesional skin from HS patients had significantly increased levels of ILC1, ILC2 and ILC3 compared to controls (42). Several studies have also identified HS lesions to be enriched in NK cells, another ILC subset, and it has been proposed that these NK cells promote sinus tract formation via release of IL-8 and cytolytic enzymes (43, 44).

Monocytes, along with their tissue-associated macrophage counterparts, also contribute to HS progression. Abu Rached et al. (2023) found that there was a significant increase in serum monocyte levels with increasing Hurley severity of HS (45). In similar work by Hunger et al. (2008), macrophages were found to be among the most common immune infiltrates in HS lesions (46), and another study also found that dermal macrophages in HS lesions expressed IL-12 and IL-23 which could upregulate differentiation of Th1 and Th17 respectively (23, 47). Expression of pro-inflammatory M1 macrophage genes were also upregulated in HS (48).

Finally, dysregulation of neutrophil activity has been implicated in HS, with neutrophil extracellular trap (NET) production as a key mechanism. NETs are web-like structures secreted by neutrophils that promote inflammation and eliminate pathogens but when dysregulated, can contribute to tissue damage (49). van Dalen et al. (2025) reported that HS patients had elevated NETs in lesional and perilesional skin, with increasing serum NET markers correlating with more severe disease (50). Additionally, various raised inflammatory mediators (eg. IL-17, IL-1 etc.) in the inflammatory environment of HS have been shown to induce neutrophil migration into HS lesions (49, 51).

Considering these above findings, TL1A/DR3-mediated regulation of innate immune cell activity may represent a key reason for anti-TL1A therapy efficacy.

DCs frequently act as a proponent of TL1A, allowing them to stimulate the TL1A/DR3 axis for the upregulation and differentiation of immune cells (31, 32). Studies such as that by Meylan et al. (2009) even suggest that DCs may be the primary source of TL1A for T cell stimulation (52). Additionally, although DCs are not typically known to express DR3, one study found that TL1A could also increase DC production of pro-inflammatory cytokines such as TNF-α and DC migration via upregulation of surface chemokine receptors (53). Inhibiting TL1A may therefore be effective in reducing DC capacity to promote inflammation in HS.

TL1A has also been shown to increase ILC3 expression of co-stimulatory molecule OX40L that upregulates T cell inflammation (54). To mimic interference of the TL1A/DR3 axis, ILC3-specific deletion of DR3 was also tested, and this was associated with reduced T cell-driven colitis (54). The TL1A/DR3 axis has been associated with increased ILC2 proliferation and secretion of pro-inflammatory IL-5/IL-13 cytokines, as well as increased NK cell secretion of IFN-γ with IL-12 and IL-18 mediation (55, 56).

Monocytes and macrophages too can express both TL1A and DR3, enabling them to act both as effectors and downstream targets. As TL1A expressors, particularly in the presence of immune complexes and inflammatory disorders, they promote activation of other DR3-expressing immune cells and production of pro-inflammatory cytokines (17, 57, 58). As DR3 expressors, TL1A has been found to stimulate macrophage differentiation into its M1 subtype (59). Inhibition of TL1A/DR3 macrophage and monocyte activity therefore represents another therapeutic strategy in HS.

Though neutrophils are also not typical producers of DR3, Perks et al. (2016) found that deleting DR3 in mice challenged with intraperitoneal *S. epidermis* supernatant injections reduced neutrophil infiltration of the peritoneum (60). The authors propose that the TL1A/DR3 axis controls production of neutrophil chemoattractants such as CXCL1, where its inhibition reduces neutrophil infiltration (60). Another indirect mechanism could be via DR3 knockdown, reducing ILC3 production of GM-CSF, another cytokine involved in neutrophil migration (61). These findings collectively indicate anti-TL1A could similarly attenuate neutrophil activity in HS.

## 3 A secondary approach to reducing fibrosis and tunnel formation

Apart from inflammation, fibrotic scarring and painful tunnel formation constitute a key physical and mental distress for HS patients. Fibroblasts are thought to alter the extracellular matrix (ECM), which results in tissue destruction and tunnel formation via production of ECM remodeling effectors such as matrix metalloproteinase 8 (62, 63). In a study by Flora et al. (2023), they also found that tissue obtained from HS lesions, including epithelized tunnels and areas of scarring, showed significantly greater pro-fibrotic fibroblast gene expression compared to non-HS lesions (63, 64). Another study also found that fibroblasts produced pro-inflammatory cytokines IL-6 and IL-33 that could act on tunnel tract keratinocytes and result in tunnel inflammation and persistence (62).

Recent evidence suggests that the TL1A/DR3 axis stimulates the pro-fibrotic activity of fibroblasts, raising the possibility that anti-TL1A therapies could mitigate fibrosis and tunnel tract formation in HS. TL1A mediates intestinal fibroblast expansion and pro-fibrotic activity, while inhibition of the TL1A/DR3 axis was capable of reducing fibroblast activity and intestinal fibrosis in mice models of chronic intestinal inflammation (65, 66). Another study by Huang et al. (2026) reported that their own anti-TL1A antibody (GB20-5A8-31) reduced bowel fibrotic area in mice models of IBD (67). Although the reduction did not reach statistical significance, this may reflect the acute nature of the mice models used, which lacked sufficient long-term fibrotic remodeling characteristic of human disease. Whether these signaling pathways in intestinal inflammation remain conserved and translate to meaningful progression in HS remains to be elucidated.

## 4 A novel triple-pronged approach to disease and comorbidities

Current evidence suggests that anti-TL1A therapy may show promise in addressing cardiometabolic risk factors. In a series of mice studies by Tougaard et al. in 2014 and 2020, TL1A deficiency conferred improvements in multiple aspects of metabolic syndrome — reduced weight gain and adipose deposition, along with increased sensitivity to insulin and glucose tolerance, were just some of the effects observed (68, 69). Maixner et al. (2020) also observed a similar trend where TL1A caused insulin resistance in adipocytes and increased lipid deposition in macrophages *in vitro* (70). A phase 1b study is also currently underway to evaluate the use of afimkibart, another anti-TL1A therapy, in the treatment of metabolic dysfunction-associated steatohepatitis (MASH) fibrosis, a commonly seen metabolic comorbidity in HS patients (71).

Additionally, elevated plasma TL1A levels were reported in patients with coronary artery disease (CAD) compared to those without (72). One proposed mechanism is that the TL1A/DR3 axis promotes atherogenesis by increasing the formation of macrophage foam cells — in an *in vitro* study by McLaren et al. (2010), they showed that TL1A caused an imbalance in macrophage uptake and efflux of cholesterol, resulting in increased lipid deposition in the macrophage cytoplasm (16). Inhibition of the TL1A/DR3 axis by anti-TL1A therapy may therefore reduce mortality from atherosclerotic diseases such as CAD or cerebrovascular accidents, though this remains largely speculative as current evidence is limited to pre-clinical models.

Finally, due to the nature of the TL1A/DR3 axis being one that involves multiple immune cell types, anti-TL1A therapy could have the added advantages of being able to target concurrent immune-mediated diseases associated with HS, namely IBD, axial spondyloarthritis (AS) and rheumatoid arthritis (RA) (73, 74). Tulisokibart and other anti-TL1A therapies, afimkibart and duvakitug, have been shown to be effective in treating IBD in recently published clinical trial results and conference abstracts (10–12, 75–77). Elevated serum levels of TL1A have also been observed in patients with AS and RA, and clinical trials are already being carried out to determine the efficacy of tulisokibart for the treatment of these diseases (78, 79).

HS is being increasingly recognized as a systemic inflammatory disorder with cardiovascular comorbidities such as metabolic syndrome, elevated cardiovascular risk and other autoimmune conditions (73, 74). Concurrent management of comorbidities is essential in improving morbidity and mortality outcomes in HS patients. Should existing pre-clinical observations translate to human disease, TL1A blockade could confer a unique advantage of addressing both the cutaneous and extracutaneous inflammatory features of HS.

## 5 Discussion and future directions

The upstream nature of the TL1A/DR3 axis may grant anti-TL1A therapies such as tulisokibart an edge over existing biologics such as adalimumab, currently the only approved anti-TNF-α biologic for management of HS (80). Unlike adalimumab, which targets TNF-α, a downstream inflammatory cytokine with limited influence over other inflammatory pathways, the inhibition of a comparatively upstream axis by tulisokibart and other anti-TL1A therapies allows for the attenuation of a wider array of immune pathways. This coordinated multi-target immunomodulation delivers a broad-reaching form of therapy that is particularly advantageous in the context of complex inflammatory diseases like HS, where interdependent inflammatory mediators often drive redundancy. Monospecific standard biologics can lead to treatment failure if parallel dysregulated pathways remain unchecked.

Additionally, the broad-reaching mechanism of action of anti-TL1A is particularly well-suited to HS, where the pathogenesis has yet to be fully elucidated (19). This enables good clinical outcomes and early remission despite incomplete knowledge of the pathophysiology of HS. This strategy enables interim disease control while deeper insights into HS pathogenesis inform development of more targeted therapies. The potential for anti-TL1A therapy modification to achieve greater clinical efficacy is another selling point; an abstract presented at the 21^st^ European Crohn’s and Colitis Organization Congress by Osterman et al. (2026) reported that their anti-TL1A therapy XmAb942 with ablated Fc-mediated effector function and Xtend™ half-life extension technology had a greater target binding and prolonged terminal half-life (estimated 71 days) in comparison to previous anti-TL1A therapies, supporting a 12 weekly dosing interval in phase 2b for maintenance therapy (81).

Nevertheless, further research is still required to accelerate the development of anti-TL1A therapy for HS. Firstly, the absence of validated animal models for preclinical studies is a critical barrier (82). Much of the perceived potential for anti-TL1A therapy in HS treatment is based on extrapolated *in vitro* studies and observations from other inflammatory disorders. Without robust animal models, it will be challenging to assess the validity of these extrapolations when applied to HS due to the fundamental differences in the immune microenvironment across different inflammatory diseases.

Another knowledge gap is the lack of a direct link between TL1A and HS development. To establish causality, validated animal models with a knockdown of TL1A or DR3 could be established to assess HS progression following interruption of the TL1A/DR3 axis. Finally, extension studies could investigate whether TNFSF15 polymorphisms, the gene encoding TL1A, are associated with changes in HS susceptibility, thereby informing the therapeutic relevance of tulisokibart and other anti-TL1A agents in HS.

Finally, although pre-clinical evidence strongly suggests that anti-TL1A therapies may be capable of targeting extra-cutaneous comorbidities of HS, there is a lack of clinical evidence to show the translatability of this effect between animal and human models. Clinical trials assessing the impact of a TL1A blockade on such extra-cutaneous comorbidities are therefore necessary to confirm our proposed triple-pronged approach.

## 6 Conclusion

Tulisokibart, afimkibart and duvatikug are anti-TL1A monoclonal antibodies under evaluation in phase 2b and 3 clinical trials for immune-mediated inflammatory diseases. Tulisokibart is currently the only anti-TL1A agent on trial for HS. These agents target the proximal TL1A/DR3 axis, which is central to HS pathogenesis, modulating both the immune and fibrotic component of HS while potentially addressing immune-mediated comorbidities such as IBD, AS and RA.
